# MCL-1 antagonism enhances the anti-invasive effects of dasatinib in pancreatic adenocarcinoma

**DOI:** 10.1038/s41388-019-1091-0

**Published:** 2019-11-18

**Authors:** Lesley Castillo, Adelaide I. J. Young, Amanda Mawson, Pia Schafranek, Angela M. Steinmann, Danielle Nessem, Ashleigh Parkin, Amber Johns, Angela Chou, Andrew M. K. Law, Morghan C. Lucas, Kendelle J. Murphy, Niantao Deng, David Gallego-Ortega, Catherine E. Caldon, Australian Pancreatic Cancer Genome Initiative (APGI), Paul Timpson, Marina Pajic, Christopher J. Ormandy, Samantha R. Oakes

**Affiliations:** 1 Cancer Research Division, Garvan Institute of Medical Research and the Kinghorn Cancer Centre, 384 Victoria Street, Darlinghurst, NSW 2010 Australia; 2 St. Vincent’s Clinical School, UNSW Medicine, 384 Victoria Street, Kensington, NSW 2052 Australia; 3 University of Sydney, Camperdown, NSW 2006 Australia

**Keywords:** Pancreatic cancer, Targeted therapies, Apoptosis

## Abstract

Pancreatic ductal adenocarcinoma (PDAC) remains one of the deadliest malignancies. It is phenotypically heterogeneous with a highly unstable genome and provides few common therapeutic targets. We found that MCL1, Cofilin1 (CFL1) and SRC mRNA were highly expressed by a wide range of these cancers, suggesting that a strategy of dual MCL-1 and SRC inhibition might be efficacious for many patients. Immunohistochemistry revealed that MCL-1 protein was present at high levels in 94.7% of patients in a cohort of PDACs from Australian Pancreatic Genome Initiative (APGI). High MCL1 and Cofilin1 mRNA expression was also strongly predictive of poor outcome in the TCGA dataset and in the APGI cohort. In culture, MCL-1 antagonism reduced the level of the cytoskeletal remodeling protein Cofilin1 and phosphorylated SRC on the active Y416 residue, suggestive of reduced invasive capacity. The MCL-1 antagonist S63845 synergized with the SRC kinase inhibitor dasatinib to reduce cell viability and invasiveness through 3D-organotypic matrices. In preclinical murine models, this combination reduced primary tumor growth and liver metastasis of pancreatic cancer xenografts. These data suggest that MCL-1 antagonism, while reducing cell viability, may have an additional benefit in increasing the antimetastatic efficacy of dasatinib for the treatment of PDAC.

PDAC is the 8th most common cause of cancer death worldwide accounting for approximately 430,000 deaths in 2018, being one of the most lethal cancers and exhibiting an mortality to incidence ratio of 94% [1]. An in-depth characterization of the pancreatic cancer genomic landscape [2–4] has revealed great heterogeneity among PDACs where highly penetrant variants are rare. The translation of this genomic information into clinical benefit remains a significant challenge [5] and there is desperate need to identify new treatments that improve the outcomes of patients suffering PDAC. In spite of the genomic heterogeneity observed in PDAC, the nonreceptor tyrosine kinase SRC is present at high levels in most PDAC specimens and pancreatic cancer cell lines. A high level of its activated form (phosphorylated on Y416) is predictive of poor outcome among low-grade pancreatic tumors [6, 7]. SRC is a member of the SRC family kinases (SFK) with pleotropic roles in the growth, survival, and invasion of pancreatic cancer [8] and suppression of SRC activity by dasatinib slows the growth of PDAC models in vitro and in vivo [9, 10]. Unfortunately the promise of these preclinical models has not been realized in clinical trials of metastatic PDAC, where single agent SFK inhibitors alone or in combination with gemcitabine showed no clinical benefit in the adjuvant setting [11–13]. Other combinatorial approaches show better activity with the triple combination of dasatinib, erlotinib (an EGFR inhibitor) and gemcitabine resulting in stable disease in ~70% of patients with tolerable safety profiles [14]. Thus the activity of agents targeting SRC may be improved with other targeted therapies that enhance its activity.

Antagonizing Myeloid cell leukemia 1 (MCL-1) in triple negative breast cancer (TNBC) can enhance the efficacy of SFK inhibitors [15]. MCL-1 is a member of the BCL-2 family of proteins that regulate the intrinsic (mitochondrial) apoptotic cascade, and a mediator of survival in both healthy and cancerous tissues [16]. MCL-1 protein levels correlate with outcome, tumor grade and therapeutic resistance in many cancers including those of the hematopoietic system, breast, lung, and pancreas [17–21]. In preclinical models of TNBC, we showed that MCL-1 modulated metastatic progression via two possible mechanisms; firstly via modulating the output of SFKs and the secondly via direct regulation of Cofilin. Cofilin is a cytoskeletal remodeling protein that is regulated by SRC activity [22, 23] and essential for actin remodeling during cellular invasion [24, 25]. As MCL-1 regulated the activity of Cofilin and the output of the SFKs in breast cancer cells, this led us to discover that drugs that antagonize MCL-1 can sensitize TNBC cells to dasatinib and suppress metastatic progression [15].

As both SRC and MCL-1 are important in the etiology of multiple cancers [26, 27], we used publicly available data to identify additional cancer contexts where a combined SRC and MCL-1 inhibitor strategy may be effective, identifying PDAC as possibly responsive to a dual SRC and MCL-1 inhibitor therapeutic strategy. We then utilized patient-derived pancreatic cell lines and orthotopic xenografts from the APGI to examine whether a dual MCL-1 and SRC inhibitor strategy was an effective antimetastatic in PDAC.

We first explored the mRNA expression of MCL1, SRC, and Cofilin1 (CFL1) across cancers in the TCGA and Australian Pancreatic Genome Initiative (APGI) to identify contexts where a dual MCL-1, and SRC inhibitor strategy may be effective. Interrogation of the TCGA datasets using cBioPortal indicated that MCL1, SRC and CFL1 are expressed among cholangiocarcinomas and PDACs to a similar extent to that of invasive breast carcinomas (Fig. 1a). Immunohistochemistry using an antibody to human MCL-1 on a tissue microarray cohort of 228 pancreatic cancers (including 188 PDACs, 20 intraductal papillary mucinous neoplasms with invasion and other mixed subtypes) from the APGI revealed a large proportion (94.7%) of PDACs and (90%) of intraductal papillary mucinous neoplasms with invasion expressed high levels of MCL-1 by IHC consistent with previous reports [28] (Supplementary Table 1 and Supplementary Fig. 1).

**Fig. 1:**
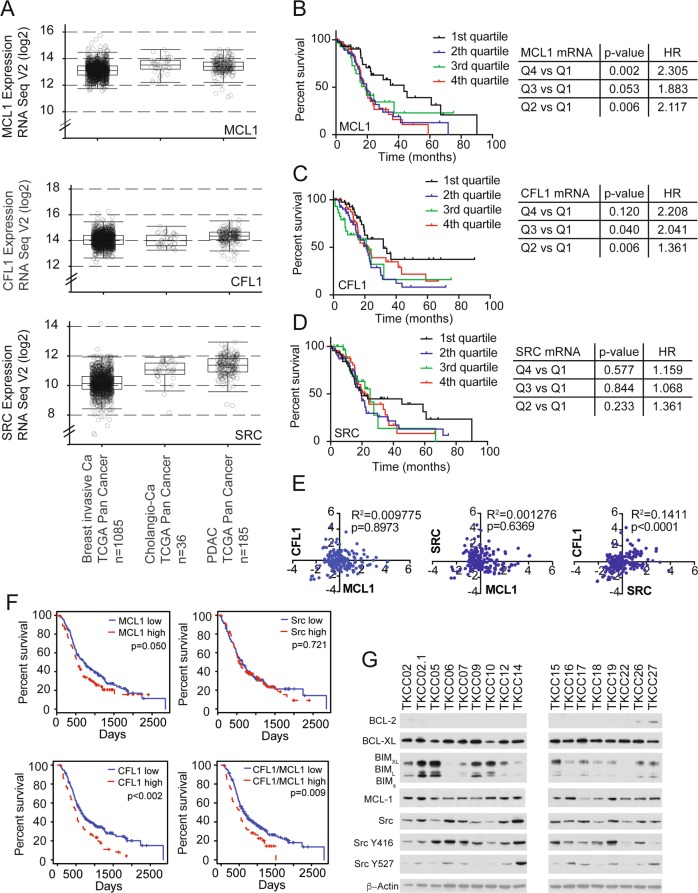
**a** Box and whisker graphs of MCL1, SRC, and Cofilin1 (CFL1) mRNA expression across breast invasive carcinoma (*n* = 1085), cholangiocarcinoma (cholangio-Ca) (*n* = 36), pancreatic adenocarcinoma (*n* = 185) among the TCGA cohort. **b** Kaplan Meier survival curves of MCL1 **c** CFL1, **d** SRC mRNA expression split by quartiles in the TCGA PDAC cohorts (*n* = 185). **e** mRNA correlation of MCL1 mRNA vs. CFL1 (left panel) and SRC (middle panel) as well as CFL1 vs. SRC (right panel). **f** Kaplan Meier survival curves of MCL1 (top left panel), CFL1 (bottom left panel), SRC (top right panel) and combined MCL1 and CFL1 mRNA expression split by quartiles in the APGI cohort (*n* = 247). Log Rank-*p*-value and hazard ratios indicated. **g** Western blots of BCL-2, BCL-XL, BIM, MCL-1, total SRC, Y416 SRC, Y527 SRC, and beta ACTIN among pancreatic cancer cells derived from the APGI cohort

To explore the clinical significance of MCL1, CFL1, and SRC in PDAC, Kaplan Meier survival analysis was performed using the mRNA expression quartiles of each gene from a total of 185 PDAC patients in the TCGA dataset. This analysis revealed that, although widely expressed among PDACs, when compared to the lowest levels of MCL1 in quartile 1, the quartiles with higher MCL1 mRNA expression were associated with worse overall survival in PDAC (Fig. 1b). A similar and significant pattern was observed using CFL1 mRNA expression quartiles (Fig. 1c), although the highest compared to the lowest quartiles failed to reach significance. SRC mRNA expression quartiles were not predictive of outcome in this cohort (Fig. 1d). There was no association of MCL1 mRNA expression with either CFL1 or SRC but we observed a significant positive correlation of SRC mRNA with CFL1 mRNA (Fig. 1e). We confirmed the observations made in the TCGA databases using data obtained from 247 PDAC patients with gene expression data from the APGI. Clinicopathological information for this cohort is provided Supplementary Table 2 and in Bailey et al. [4]. This analysis showed that the highest levels (top 25% vs. lowest 25%) of both MCL1 and CFL mRNA correlated with worse overall survival (Fig. 1f, left panels). The mRNA expression of SRC showed no prognostic power (Fig. 1f top right panel). When used together, top quartile levels of both MCL1 and CFL1 were predictive of worse outcome when compared to lower quartile levels in the APGI (Fig. 1f bottom right panel). Western blotting showed that activated SRC (Y416) was a feature among a panel of patient-derived pancreatic cancer cell lines (Fig. 1g). The BH3 only pro-apoptotic and MCL-1 interacting protein BIM was variable across each line. Furthermore the majority of PDACs were MCL-1 and BCL-XL positive but BCL-2 negative potentially indicating a preference on either MCL-1 or BCL-XL for survival.

As the TKCC05 PDAC patient-derived cell line showed high levels of MCL-1, BIM and total and pSRC levels, this line was selected to examine the efficacy of a dual MCL-1 and SRC inhibitor strategy. This line can also invade into 3-dimensional collagen I matrices and successfully engraft as orthotopic xenografts in immune-compromised mice, spread to the liver and other organs providing a useful model of pancreatic metastasis [29]. Increasing concentrations of the MCL-1 antagonist S63845 resulted in elevated levels of MCL-1 similar to what was observed when human breast cancer cell lines MDA-MB-231 and MDA-MB-468 were treated with S63845 for 48 h (Fig. 2a) [15, 30]. Treatment with 500 nM S63845 produced a significant suppression of total Cofilin, which was maintained over a 72-h period (Fig. 1b, c) and also resulted in a trend towards an increased ratio of serine 3 (S3) phosphorylated (inactivated) Cofilin to total Cofilin at 24 h post treatment (Fig. 2d). MCL-1 antagonism did not alter the levels of total SRC but decreased the ratio of Y416 phosphorylated (activated) SRC to total SRC over the entire 72 h period suggestive of reduced activity (Fig. 2e). Bliss synergy analysis showed that the combination of S63845 and dasatinib (0–25 µM) was synergistic across a wide range of concentrations at 48 h and 72 h post treatment (Fig. 2f).

**Fig. 2:**
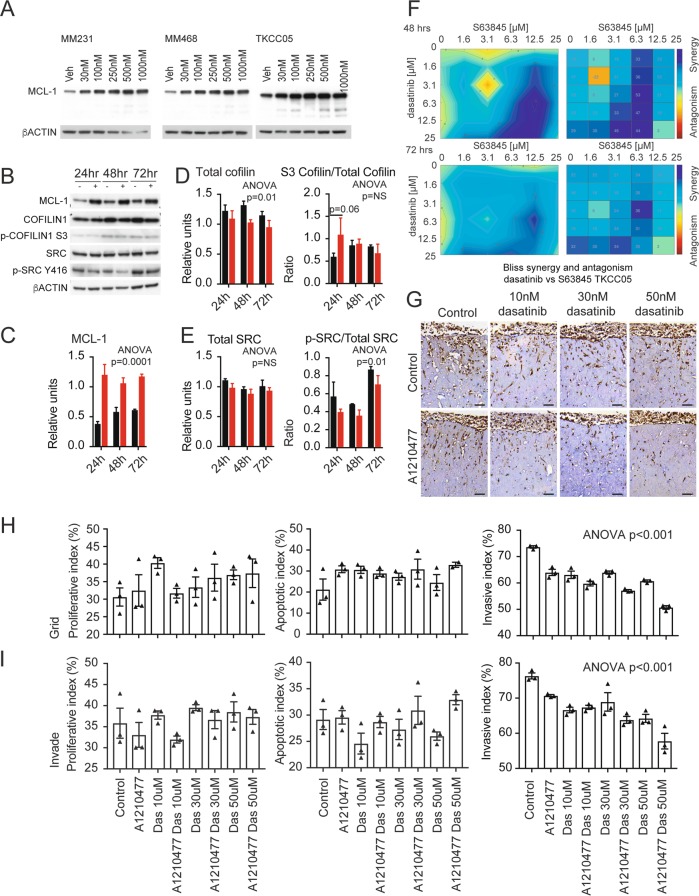
**a** Western blots of MCL-1 and beta ACTIN from MDA-MB-231, MDA-MB-468 breast cancer and TKCC05 pancreatic cancer cells treated with increasing concentrations of S63845. **b** Western blots and densitometry quantification of MCL-1 (**c**), total Cofilin (**d**, left panel), ratio of S3 phosphorylated Cofilin/total Cofilin (**d**, right panel), total SRC (**e**, left panel), the ration of Y416 phosphorylated SRC to total SRC (**e**, right panel) from TKCC05 pancreatic cancer cells treated with 250 nM S63845 over a 72 h period and normalized to beta ACTIN. *N* = 4 independent experiments, error bars, unpaired *t*-tests between groups and two-way ANOVA for treatments (vehicle vs. S63845) indicated. **f** Bliss synergy contour plot (left panels) and synergy matrix (right plots) of TKCC05 pancreatic cancer cells treated with increasing concentrations (0–25 µM) of S63845 and dasatinib at 48 h (upper panels) and 72 h (lower panels). **g** Representative immunohistochemistry using an antibody to human Vimentin on TKCC05 pancreatic cancer cells invading into fibrillar Collagen I organotypic matrices and treated with the indicated concentrations of A1210477 and dasatinib (**h**, **i**). Bar graphs showing the quantification of Ki67 (proliferating cells, left panels), cleaved caspase 3 (apoptotic cells, middle panels) and Vimentin (invasion index, right panels) of TKCC05 pancreatic cancer cells treated with the indicated concentrations of A1210477 and dasatinib at seeding (upper panels, grid) or 5 days after seeding (lower panels, invade). Error bars and two-way ANOVA *p*-value between treatments indicated

We then examined the effects of MCL-1 or SFK antagonism alone and in combination in three-dimensional fibrillar Collagen I matrices in vitro (Fig. 2g [31]). There were no significant effects of SRC inhibition by dasatinib or MCL-1 antagonism by A1210477 alone or in combination on proliferation or apoptosis as measured by Ki67 and cleaved caspase 3 immunohistochemistry respectively (Fig. 2h, i). However, there was a trend towards enhanced apoptosis when S63845 was combined with dasatinib when administered 5 days post exposure to an air-liquid interface. This was after when they had begun to invade, mimicking the clinical presentation of this disease, which often is associated with local invasion. In contrast dasatinib treatment resulted in a significant and dose dependent decrease in the ability of TKCC05 cells to invade through the organotypic matrix. Treatment with A1210477 similarly reduced their invasive capacity and significantly enhanced the effects of dasatinib across the dosage range equally when the drugs were administered just after seeding (Fig. 2h right panel) and after when they had begun to invade (Fig. 2i, right panel).

We next investigated whether dual inhibition of MCL-1 and SRC would be effective in the treatment of PDACs in vivo (Fig. 3). TKCC05 patient-derived pancreatic cells were implanted directly in the pancreas of immune-compromised NODScidIL2gamma^–/–^ mice and bioluminescent imaging was used confirm successful engraftment and monitor the growth and spread of TKCC05 patient-derived pancreatic xenografts over 5 weeks (Fig. 3a). The rate of expansion of primary pancreatic tumors was not significantly different between mice treated with vehicle, S63845, dasatinib or a combination (Fig. 3b) but we observed a small but significant reduction in the weight of the primary tumor at 5 weeks post implantation (Fig. 3c). There were no effects of the single agents on primary tumor proliferation and apoptosis as measured by Ki67 and cleaved caspase 3 immunohistochemistry respectively, but a small and significant decrease in proliferation was observed in response to combination treatment (Fig. 3d, e). Bioluminescent imaging at 5 weeks post surgery suggested that the combination with S63845 and dasatinib reduced the spread of the TKCC05 patient-derived pancreatic xenografts (Fig. 3a). Immunohistochemistry using an antibody to human MCL-1 in resected PDAC tumors from this model revealed both nuclear and cytoplasmic staining (Supplementary Fig. 2A). Treatment with S63845 produced a significant increase in MCL-1 intensity (Supplementary Fig. 2B) consistent with S63845 extending MCL-1 protein half-life levels and providing a biomarker of response [30]. Both the lungs and livers of mice bearing TKCC05 patient-derived pancreatic xenografts were collected at 5 weeks and stained with an antibody against human vimentin to highlight disseminated PDAC cells [32] (Fig. 3f–i). We observed far fewer metastases in the lungs compared to the livers at this time point. While no effect of any treatment was detected in the lungs of these mice (Fig. 3g), the combination of S63845 and dasatinib produced a significant reduction in liver metastasis compared to vehicle and single agent therapy (Fig. 3i).

**Fig. 3:**
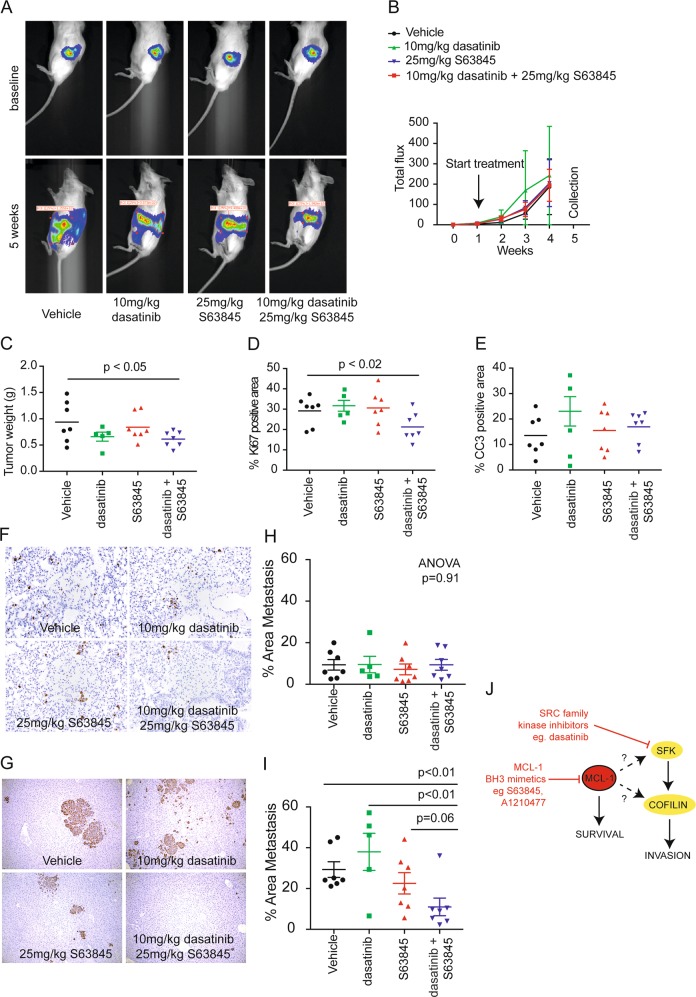
**a** Representative bioluminescent images of mice bearing TKCC05 pancreatic cancer xenografts at surgery (baseline) or at 5 weeks after surgery (5 weeks) and treated with vehicle (*n* = 7), 25 mg/kg S63845 (*n* = 7), 10 mg/kg dasatinib (*n* = 5, 2 were excluded from the dasatinib cohort as they reached ethical end point one week early due to ascites) or combined S63845 and dasatinib (*n* = 7). **b** Line graphs of the average bioluminescence of mice bearing TKCC05 pancreatic cancer xenografts at surgery (baseline) over a 5 week period treated with vehicle, 25 mg/kg S63845, 10 mg/kg dasatinib or combined S63845 and dasatinib. Dot plots of **c** tumor weight, **d** tumor Ki67 positivity (**e**) and cleaved caspase 3 positivity in TKCC05 pancreatic cancer orthotopic primary tumors. Representative photomicrographs taken at ×20 objective of the **f** lungs and **g** livers from mice bearing TKCC05 pancreatic cancer xenografts subjected to immunohistochemistry using an antibody against vimentin and (**h**) dot plots showing the average area of metastasis in the lungs and (**i**) livers of mice bearing TKCC05 pancreatic cancer xenografts at 5 weeks post surgery (each dot is average of 15 images within one mouse). Unpaired *t*-tests between groups and one-way ANOVA *p*-value for treatments (vehicle vs. S63845) illustrated. **j** Model schematic of MCL-1 and SRC regulation of Cofilin. Combined inhibition of MCL-1 by BH3 mimetics such as S63845 and A1210477 can enhance the anti-invasive effects of dasatinib via a possible direct or indirect regulation of Cofilin via SRC

Here we have shown that MCL-1, Cofilin, and SRC are widely expressed among PDACs (Fig. 1) with high MCL-1 protein levels detected among 94.7% of all PDACs in the APGI tissue microarray cohort. Elevated expression of MCL1 resulted in a two-fold higher risk of death when compared to patients with the lowest quartile mRNA expression of MCL1 in the TCGA and APGI cohorts (Fig. 1a, f respectively). Similar observations were true for Cofilin in both the TCGA (Fig. 1c) and the APGI cohorts (Fig. 1f), although there was no significant difference between CFL1 low group and CFL1 high group in the TCGA cohort (Fig. 1c). Possible reasons for this discrepancy could be the methodology in assessing mRNA expression (RNAseq in TCGA vs. array based gene expression in the APGI) as well as a greater number of patients analyzed in the APGI cohort (247) vs. the TCGA cohort (185), reaching significance in the APGI cohort. As Cofilin is tightly linked to SRC activity [22, 23], and we have shown can be regulated by MCL-1, these data suggest that up to 75% of patients with PDACs may benefit from a combinatorial MCL-1 and SFK inhibitor strategy. A similar benefit could be possible for patients with cancers dependent on MCL-1 and SFK activity via Cofilin e.g. cholangiocarcinomas, but this remains to be investigated (Fig. 1a). Furthermore, as 75% of PDACs contain inactivating mutations in TP53 [33], it is accepted that these tumors are likely to have an intact apoptotic cascade and therefore sensitive to antagonism by BH3 mimetics [34]. We have shown that MCL-1 antagonism can potently sensitize PDACs to SFK inhibition by dasatinib, and that MCL-1 protein levels as measured by immunohistochemistry could be used as a biomarker for response. The importance of SFK in pancreatic cancer is widely recognized [35–37], hence there has been extensive research into the development of agents that target the SFK in the clinical setting. Unfortunately the promise of preclinical experiments has been met with disappointment in clinical trials with single agent dasatinib [11], and Phase II clinical trials of dasatinib or saracatinib in combination with gemcitabine failing to show any clinical benefit in patients with refractory PDAC [12, 13]. A more recent combination shows better activity with the triple combination of dasatinib, erlotinib (an EGFR inhibitor) and gemcitabine resulting in stable disease in ~70% of patients with tolerable safety profiles [14]. Interestingly this combination includes an agent that antagonizes EGFR, a key growth factor that controls MCL-1 transcription [38], possibly suggesting that the success of this trial could be at least, in part, due to the effects of erlotinib on EGFR driven MCL-1 transcription.

We have previously shown that in the MDA-MB-231 TNBC cell lines in culture, the effects of MCL-1 are largely limited to its anti-invasive effects possibly via its regulation of the cytoskeletal remodeling protein Cofilin and/or by the SFKs [15]. Similarly in PDAC cancer cells, S63845 also significantly modulated the expression of Cofilin and the Y416 phosphorylated and activated form of SRC. In addition, the effects of the S63845 antagonist in combination with dasatinib were predominantly restricted to outcomes of cellular invasion (Fig. 2) and metastasis (Fig. 3). These results suggest that MCL-1 modulation of metastatic progression via SRC or Cofilin may be present in multiple cancer contexts. Metastatic progression requires remodeling of the cytoskeleton, dynamic membrane changes, cellular invasion and localized tissue destruction [39], and this is regulated by the SFKs and their targets, including cSRC, FYN, YES, Paxillin, Cofilin, Cortactin, Rac and Rho [27, 40]. SRC was not predictive of outcome in the TCGA or the APGI cohorts but correlated with the expression of the cytoskeletal remodeling protein Cofilin, consistent with SRC’s known regulation of this protein [22, 23]. We have also shown that MCL-1 can modulate Cofilin expression (Fig. 2). A schematic model for these observations is provided in Fig. 3h, where MCL-1, a known pro-survival protein, may directly or indirectly regulate Cofilin expression via SRC to control invasion. We have already established that high levels of MCL-1 place it in close proximity to Cofilin in breast cancer models [15]. While the full details underlying this mechanism remain to be discovered, the data presented here provide a possible explanation as to why dual antagonism of MCL-1 and SRC is synergistic.

In conclusion we have shown MCL-1 is widely expressed by and can predict outcome in PDAC. Therapeutic targeting of MCL-1 using BH3 mimetics (e.g., S63845, A1210477, ADZ5991, MIK665/S64315 etc.) is currently being investigated in clinical trials for patients with multiple myeloma, acute myeloid leukemia, and myelodysplastic syndrome (NCT02992483, NCT02979366 and NCT03672695) and may provide a way of sensitizing these tumors to dasatinib and provide a new therapeutic strategy alone or in combination with standard of care for PDAC.

## Materials and methods

All materials and methods are provided in the Supplementary information

## Supplementary information

Supplementary Materials

Supplementary Figure 1

Supplementary Figure 2

Supplementary Table 1

Supplementary Table 2

APGI membership List for Publications 2019
