# Supplementary material for: MCL-1 antagonism enhances the anti-invasive effects of dasatinib in pancreatic adenocarcinoma
Source: Oncogene. 2019 Nov 18;39(8):1821–9. doi: 10.1038/s41388-019-1091-0 (PMC7033042; doi:10.1038/s41388-019-1091-0)
Supplement: Supplementary file 1 — Supplementary Materials [file 41388_2019_1091_MOESM1_ESM.docx]

**Materials and Methods**

**Public data set, APGI data analysis and ICGC tissue microarrays**

RNAseq mRNA V2 expression plots of MCL1, SRC and CFL1 (Cofilin1) among invasive breast carcinoma, cholangiocarcinoma and pancreatic adenocarcinoma in the Cancer Genome Atlas Program (TGCA) Pan Cancer Atlas cohort were downloaded from cBioPortal ^1, 2^. PDAC survival data was obtained from the Pancreatic adenocarcinoma cohort n=185) was analysed using a Kaplan Meier Survival and log-rank test used to compare the survival distributions of two quartiles. mRNA expression data of SRC, MCL-1 and CFL1 were correlated and a Pearson’s correlation used to determine whether each was linearly correlated.

Array-based gene expression and clinical data of a second cohort of PDACs from the APGI as part of the International Cancer Genome Consortium (ICGC) (n=247) were downloaded from ICGC data portal (<https://dcc.icgc.org/projects/PACA-AU>). Kaplan Meier analysis was performed by comparing the survival outcome of patients stratified by top quartile versus the remainder using MCL1, SRC, CFL1 mRNA expression. A subset of these samples from resected pancreatic ductal adenocarcinomas were obtained from patients the APGI cohort and made into tissue microarrays. All approvals for the use of the use of the APGI Bioresource (tissue microarrays) for MCL-1 IHC were obtained via the New South Wales Pancreatic Network and The Australian Familial Pancreatic Cancer Cohort (collectively the APGI). These included a total of 228 pancreatic cancers (including 188 pancreatic ductal adenocarcinomas, 20 intraductal papillary mucinous neoplasms with invasion and other mixed subtypes, Supplementary Table 1) ^3-5^. Tissue samples were fixed in 4% formalin, embedded in paraffin and 96 spot tissue microarrays containing triplicate cores were stained with MCL-1 (Invitrogen AHO0102 Clone RC13) using Leica BOND immunohistochemistry as per the manufacturer’s instructions. The complete APGI tissue microarray cohort was scored by an expert pathologist (AC), where most pancreatic cancers showed nuclear and cytoplasmic staining with scoring categorised into positive staining (2), weak staining (1) or negative staining (0). All ethical approvals for the use of ICGC APGI samples was approved by the relevant authorities.

**The Kinghorn Cancer Centre (TKCC) PDAC orthotopic xenograft and immortalised cell lines**

Eleven patient derived cell lines (TKCC02-27, Figure 1G) were derived from patient-derived tumour obtained by the APGI ^3^ and were established as previously described ^6^. TKCC-05 were maintained patient derived cell lines in a 1:1 mixture of Dulbecco's modified Eagle's medium and Ham's F12 medium (Thermo Fisher Scientific, AU) supplemented with fetal bovine serum (7.5%), 15mM HEPES (Thermo Fisher Scientific, AU), 1.2% Glucose 25ug/mL apo-transferrin, 40ng/ml hydrocortisone (Sigma-Aldrich, US), 0.1IU/mL insulin (Novo Nordisk, DK) and 10ng/ml human recombinant epidermal growth factor. All cell lines were routinely tested for mycoplasma contamination.

***In vitro* experiments**

MDA-MB-231 and MDA-MB-468 cell lines, obtained from American Type Culture Collection and maintained in Roswell Park Memorial Institute (RPMI) 1640 Medium (Thermo Fisher Scientific) supplemented with fetal bovine serum. MDA-MB-231 and MDA-MB-468 cell lines and authenticated by STR profiling prior to the experiments being completed. MDA-MB-231, MDA-MB-468 and TKCC05 patient derived cells lines were maintained as above and exposed to vehicle or increasing concentrations of S63845 (30nM to 1000nM) and harvested at 48hrs and assayed for MCL-1 levels by western blot. The effects of MCL-1 antagonism (500 nM S68345) on Cofilin1, S3 phosphorylated Cofilin1, SRC, Y416 phosphorylated SRC levels were assessed at 24-72 hours post treatment. For Bliss Synergy experiments, increasing concentrations of S63845 and dasatinib were examined using synergy calculated using a Bliss Synergy Calculation and images drawn and calculated using CalcuSyn.

**SDS-PAGE and western blot analysis**

Cells grown for 24, 48 or 72 hours were washed with PBS, and harvested by scraping into normal lysis buffer (50mM HEPES, 150mM NaCl, 10% glycerol, 1% triton X-100, 1.5mM MgCl2, 1mM EDTA, 10mM pyrophosphate, 10mM NaF) with protease and phosphatase inhibitors (ROCHE). Lysates were clarified by centrifugation and protein concentration determined via Bradford Assay. 20μg reduced protein was resolved by NuPAGE 4-12% gels (Life Technologies) in MES or MOPs gel running buffer as appropriate. Proteins were transferred onto PVDF membrane, blocked with 5% skim milk and probed overnight 4C with antibodies directed against MCL-1 (Cell Signaling Technologies (CST) 5453), BCL-2 (Santa Cruz 492), BCL-XL (CST 2764), BIM (CST 2933), SRC (CST 2123), Phospho-SRC (Tyr416) (CST 6943), Phospho-SRC (Tyr527) (CST 2105), Cofilin (CST 5175), Phospho-Cofilin (Ser3) (CST 3313) and beta-ACTIN (Sigma AC-74, A5316). Bound protein was visualized using HRP-conjugated secondary antibodies (GE Healthcare) and chemiluminesence.

**3D organotypic collagen I/fibroblast invasion assay**

The production of contracted matrices is described elsewhere ^7^. Fibroblast- contracted matrices were seeded with 1x10^5^ TKCC05 cells and allowed to grow for 4 days, mounted on a metal grid and raised to an air–liquid interface to initiate invasion, which resulted in the matrix being fed from below with the media supplemented with either vehicle, 5µM of the MCL-1 BH3 mimetic inhibitor A1210477, 10, 30 or 50µM dasatinib or combination of A1210477 and dasatinib commencing on (day 1) or 5 days (day 5) prior to matrices being exposed to the air-liquid interface. All treatments were performed on three independent matrices, per replicate. Cells were allowed to invade for a total of 21 days towards the chemo-attractive gradient created by the air–liquid interface, with media and drug refreshed three times weekly, and then harvested for immunohistochemistry for Vimentin (Leica NCL-L-VIM-V9) (invasion), Ki67 (ThermoScientific SP6) (proliferation), or Cleaved Caspase 3 (Cell Signaling Asp175 5A) (apoptosis) and scored. Nine areas of 500x500 μm were selected and averaged from 3 independent experiments as representative of the invasion, proliferation or apoptosis for each condition and each antibody. The sum of these cells, and the number of cells on the matrix surface classified as having the ‘opportunity’ to invade (Vimentin positive), was used to calculate the invasive index as a proportion of invaded cells. The proliferative index was scored as a ratio of brown (Ki67-positive cells) to blue & brown (total cells) within or above the matrix. Similarly, the apoptotic index was scored as a ratio of brown (Cleaved caspase 3-positive cells) to blue and brown (total cells) within or above of the matrix. Columns, mean; error bars represent ± SEM, scale bars: 50 μm; An analysis of variance (ANOVA) was used to examine the difference across treatments.

**Mice**

All animal ethics approvals were reviewed and approved by the Garvan Institute of Medical Research and St. Vincent's Hospital Animal Ethics Committee. Immune-compromised NODScidIL2gamma^–/–^ mice were housed in SPF conditions in a 12-hour:12-hour light:dark cycle and given food and water ad libitum. 15,000 luciferase- labelled pancreatic patient derived cell lines were injected into the pancreas of 6-8 week old female NODScidIL2gamma^–/–^ mice. Seven mice (total 28) were randomized into four treatment groups of vehicle, 25mg/kg S63845 IV administered weekly, 10mg/kg dasatinib by daily oral gavage (MON-FRI) or the combination from one-week post surgery until 5 weeks. Sample size was estimated based on the previous experience of the investigators. Mice were rested on weekends and tumour burden was monitored weekly by bioluminescent imaging and analyzed (IVIS Spectrum; Living Image Software, Perkin-Elmer, US). At 5 weeks post surgical implantation, mice were euthanized with CO_2_ asphyxiation and the pancreas and tumour (very little pancreatic normal tissue remained at 5 weeks post implantation), liver and lungs were harvested and fixed for 4 hours in 10% buffered formalin at room temperature. Harvested tissues were processed for histology and either stained with hematoxylin and eosin for routine histochemistry or stained with high molecular vimentin (Leica NCL-L-VIM-V9), MCL-1 (Invitrogen AHO0102 Clone RC13), Cleaved Caspase-3 (CST ASP175 9664) and Ki67 (ThermoScientific SP6) using DAKO or Leica BOND immunohistochemistry as per the manufacturer’s instructions. All sections from tumours and lungs in each model were cut, sectioned, retrieved and stained at the same time.

**Image analysis and statistical analysis**

Quantification of number and size of lung and liver metastasis (using coded sections stained with an antibody against ant- human-vimentin) was performed using a macros designed in our laboratory for use with FIJI image analysis available at <http://fiji.sc/Fiji> producing high-throughput, highly reproducible and standardized quantification of immunohistochemistry ^8^. For MCL-1 IHC, the macros were further optimized to measure stain intensity with intensity values binned into quartiles of expression for Chi-squared analysis. Briefly, IHC area analysis was measured by processing 20 images per mouse tumor, liver or lung digitally after acquisition by first applying a tritanope colorblind filter and then replacing red with magenta. A color deconvolution is applied using the H&E vector to produce a purple, pink, and green RGB image. The purple image is converted to 8-bit grey-scale image and threshold applied, then converted to a binary image, and processed to segment adjacent nuclei. A region of interest (ROI) was used to count and measure the area and intensity of all nuclei and then average for each biological replicate (mouse). All other data and statistics were analyzed in Prism6 for MacOSX. Data were graphed and parametric or nonparametric tests, as indicated in the figure legends, were used for normally distributed and skewed data respectively, and statistically significant groups were determined as shown in the figures.

**Supplementary References**

1 Cerami E, Gao J, Dogrusoz U, Gross BE, Sumer SO, Aksoy BA *et al*. The cBio cancer genomics portal: an open platform for exploring multidimensional cancer genomics data. Cancer discovery 2012; 2: 401-404.

2 Gao J, Aksoy BA, Dogrusoz U, Dresdner G, Gross B, Sumer SO *et al*. Integrative analysis of complex cancer genomics and clinical profiles using the cBioPortal. Science signaling 2013; 6: pl1.

3 Bailey P, Chang DK, Nones K, Johns AL, Patch AM, Gingras MC *et al*. Genomic analyses identify molecular subtypes of pancreatic cancer. Nature 2016; 531: 47-52.

4 Dreyer SB, Pinese M, Jamieson NB, Scarlett CJ, Colvin EK, Pajic M *et al*. Precision Oncology in Surgery: Patient Selection for Operable Pancreatic Cancer. Ann Surg 2018: 0000000000003143.

5 Pinho AV, Van Bulck M, Chantrill L, Arshi M, Sklyarova T, Herrmann D *et al*. ROBO2 is a stroma suppressor gene in the pancreas and acts via TGF-beta signalling. Nat Commun 2018; 9: 5083. doi: 5010.1038/s41467-41018-07497-z.

6 Chou A, Froio D, Nagrial AM, Parkin A, Murphy KJ, Chin VT *et al*. Tailored first-line and second-line CDK4-targeting treatment combinations in mouse models of pancreatic cancer. Gut 2018; 67: 2142-2155. doi: 2110.1136/gutjnl-2017-315144. Epub 312017 Oct 315128.

7 Timpson P, McGhee EJ, Erami Z, Nobis M, Quinn JA, Edward M *et al*. Organotypic collagen I assay: a malleable platform to assess cell behaviour in a 3-dimensional context. Journal of visualized experiments : JoVE 2011: e3089.

8 Law AMK, Yin JXM, Castillo L, Young AIJ, Piggin C, Rogers S *et al*. Andy's Algorithms: new automated digital image analysis pipelines for FIJI. Sci Rep 2017; 7: 15717. doi: 15710.11038/s41598-15017-15885-15716.

**Supplementary Figure 1.**

Representative photomicrographs of normal and pancreatic ductal adenocarcinoma tissue microarrays the APGI subjected to immunohistochemistry using an antibody against MCL-1 showing negative (0), weak (1) and strong staining (2). Scale bars 50µM.

**Supplementary Figure 2.**

(A) Representative photomicrographs of MCL-1 localization within TKCC05 pancreatic ductal adenocarcinoma orthotopic tumors grown in mice and treated with vehicle, 10mg/kg dasatinib, 25mg/kg S63845 and 10mg/kg dasatinib and 25mg/kg S63845 combined. MCL-1 was expressed both in the nuclei and cytoplasm of pancreatic ductal adenocarcinoma orthotopic tumors. (B) Quantification of the average intensity of MCL-1 intensity across all cells within TKCC05 pancreatic ductal adenocarcinoma orthotopic tumors treated with vehicle, 10mg/kg dasatinib, 25mg/kg S63845 and 10mg/kg dasatinib and 25mg/kg S63845 and binned into quartiles of intensity from 1^st^ quartile (lowest intensity) to the 4^th^ quartile (highest intensity). 15 images per tumor were used for MCL-1 intensity quantification with every nuclei scored and the average intensity value from each mouse binned into quartiles as a percentage of total number of mice and used for Chi squared analysis.

**Supplementary Table 1.**

MCL-1 immunohistochemical scoring of pancreatic cancers in the APGI tissue microarray cohort including samples of PDAC (n=188), intraductal papillary mucinous neoplasm with invasion (n=20), ampullary adenocarcinoma (n=2), cholangiocarcinoma (n=1), clear cell carcinoma (n=1), mucinous cystadenocarcinoma (n=1), adenosquamous carcinoma (n=7), mucinous non-cystic carcinoma (n=4), undifferentiated carcinoma with osteoclast-like giant cells (n=3) and small cell carcinoma (n=1). Table includes columns for de-identified patient IDs, raw immunohistochemical score (0: negative, 1: weak staining, 2: positive staining, X: no tissue) of 3 TMA sections from the same patient and their average, histological grade, histological type, tumor size and overall stage at resection. Summary tables of stage and percent MCL-1 positive, weak and negative are provided for those cases with 5 of more samples separated by histological type and all cases.

**Supplementary Table 2.**

Clinical characteristics of the APGI cohort. This table provides clinicopathological data for 247 patients analyzed using Illumina HT-12 expression arrays and downloaded from ICGC data portal (<https://dcc.icgc.org/projects/PACA-AU>. These patients were used in the Kaplan-Meier analysis for overall outcome of MCL1, SRC and CFL1 mRNA expression in Fig.1F.
