# Supplementary figures and images for: MCL-1 antagonism enhances the anti-invasive effects of dasatinib in pancreatic adenocarcinoma
Source: Oncogene. 2019 Nov 18;39(8):1821–9. doi: 10.1038/s41388-019-1091-0 (PMC7033042; doi:10.1038/s41388-019-1091-0)

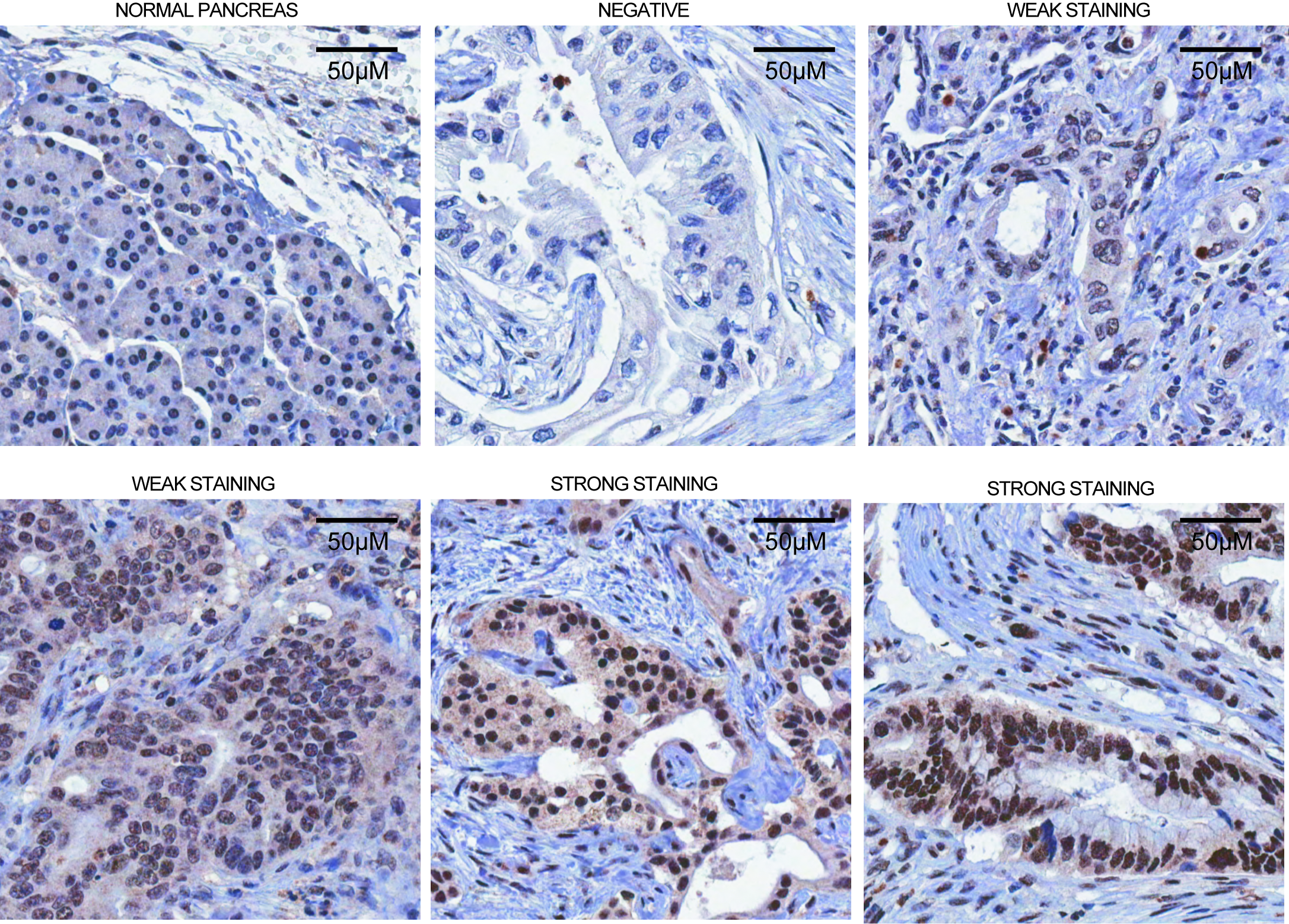

Supplement: Supplementary file 2 — Supplementary Figure 1 [file 41388_2019_1091_MOESM2_ESM.tif]

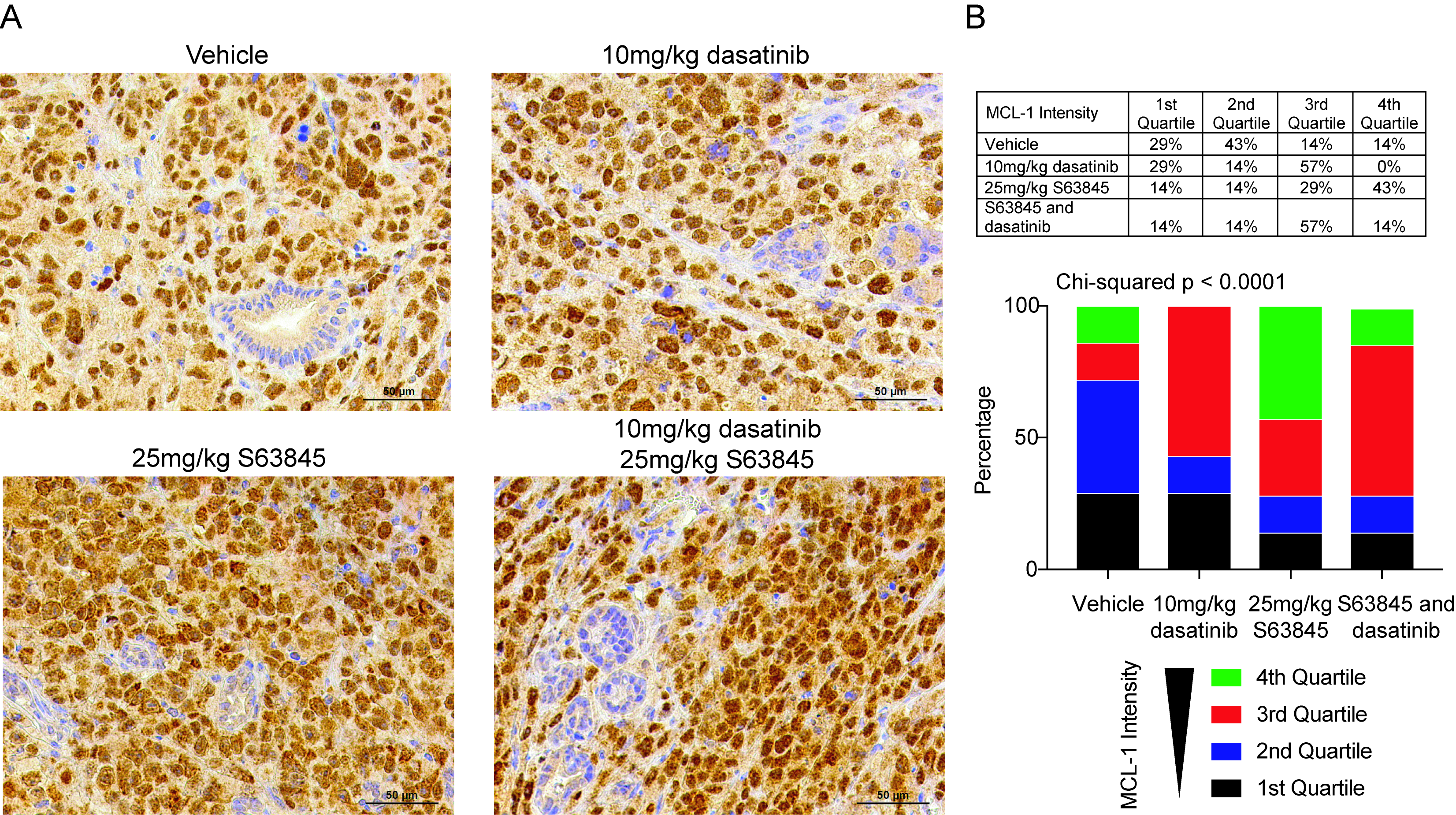

Supplement: Supplementary file 3 — Supplementary Figure 2 [file 41388_2019_1091_MOESM3_ESM.tif]
