# Supplementary material for: MCL-1 antagonism enhances the anti-invasive effects of dasatinib in pancreatic adenocarcinoma
Source: Oncogene. 2019 Nov 18;39(8):1821–9. doi: 10.1038/s41388-019-1091-0 (PMC7033042; doi:10.1038/s41388-019-1091-0)
Supplement: Supplementary file 6 — APGI membership List for Publications 2019 [file 41388_2019_1091_MOESM6_ESM.docx]

**Australian Pancreatic Cancer Genome Initiative**

**Garvan Institute of Medical Research** Amber L. Johns^1^, Anthony J Gill^1, 5^, Lorraine A. Chantrill^1,23^, Paul Timpson^1,^ Angela Chou^1,5^, Marina Pajic^1^, Angela Steinmann^1^, Mehreen Arshi^1^, Tanya Dwarte^1^, Danielle Froio^1^, Ashleigh Parkin^1^ , David Hermann^1^, Claire Vennin^1^, Thomas R Cox^1^, Brooke Pereira^1^, Shona Ritchie^1^, Daniel A Reed^1^, Cecilia R Chambers^1^, Xanthe Metcalf^1^, Max Nobis^1^, **QIMR Berghofer Medical Research Institute** Nicola Waddell^2^, John V. Pearson^2^, Ann-Marie Patch^2^, Katia Nones^2^, Felicity Newell^2^, Pamela Mukhopadhyay^2^, Venkateswar Addala^2^, Stephen Kazakoff^2^, Oliver Holmes^2^, Conrad Leonard^2^, Scott Wood^2^. **University of Melbourne, Centre for Cancer Research** Sean M. Grimmond^3^, Oliver Hofmann^3^. **University of QLD, IMB** Angelika Christ^4^, Tim Bruxner^4^. **Royal North Shore Hospital** Jaswinder S. Samra^5^, Nick Pavlakis^5,^ Hilda A. High^5^. **Bankstown Hospital** Ray Asghari^6^, Neil D. Merrett^6^, Darren Pavey^6^, Amitabha Das^6^. **Liverpool Hospital** Peter H. Cosman^7^, Kasim Ismail^7^, Chelsie O’Connnor^7^. **St Vincent’s Hospital** Alina Stoita^8^, David Williams^8^, Allan Spigellman^8^**. Westmead Hospital** Vincent W. Lam^9,^ Duncan McLeod^9^, Judy Kirk^9^. **Royal Prince Alfred Hospital, Chris O’Brien Lifehouse** James G. Kench^10^, Peter Grimison^10^, Caroline L. Cooper^10^, Charbel Sandroussi^10^, Annabel Goodwin^7,10^. **Prince of Wales Hospital** R. Scott Mead^1,11^, Katherine Tucker^11^, Lesley Andrews^11^. **Fremantle Hospital** Michael Texler^12^, Cindy Forest^12^, Krishna P. Epari^12^, Mo Ballal^12^, David R. Fletcher^12^, Sanjay Mukhedkar^12^. **St John of God Healthcare** Nikolajs Zeps^14^, Maria Beilin^14^, Kynan Feeney^14^. **Royal Adelaide Hospital** Nan Q Nguyen^15^, Andrew R. Ruszkiewicz^15^, Chris Worthley^15^. **Flinders Medical Centre** John Chen^16^, Mark E. Brooke-Smith^16^, Virginia Papangelis^16^. **Envoi Pathology** Andrew D. Clouston^17^. **Princess Alexandria Hospital** Andrew P. Barbour^18^, Thomas J. O’Rourke^18^, Jonathan W. Fawcett^18^, Kellee Slater^18^, Michael Hatzifotis^18^, Peter Hodgkinson^18^. **Austin Hospital** Mehrdad Nikfarjam^19^. **Johns Hopkins Medical Institutes** James R. Eshleman^20^, Ralph H. Hruban^20^, Christopher L. Wolfgang^20^. **ARC-Net Centre for Applied Research on Cancer** Aldo Scarpa^21^, Rita T. Lawlor^21^, Stefania Beghelli^21^, Vincenzo Corbo^21^, Maria Scardoni^21^, Claudio Bassi^21^. **University of Glasgow** Andrew V Biankin^1, 22^, Judith Dixon^22^, Nigel B. Jamieson^22^ David K. Chang^1, 22^

^1^The Kinghorn Cancer Centre, Garvan Institute of Medical Research, 370 Victoria Street, Darlinghurst, Sydney, New South Wales 2010, Australia.

^2^QIMR Berghofer Medical Research Institute, 300 Herston Rd,
Herston, Queensland 4006, Australia.

^3^University of Melbourne, Centre for Cancer Research, Victorian Comprehensive Cancer Centre, 305 Grattan Street, Melbourne, Victoria 3000, Australia.

^4^ Institute for Molecular Bioscience, University of QLD, St Lucia, Queensland 4072, Australia.

^5^Royal North Shore Hospital, Westbourne Street, St Leonards, New South Wales 2065, Australia.

^6^Bankstown Hospital, Eldridge Road, Bankstown, New South Wales 2200, Australia.

^7^Liverpool Hospital, Elizabeth Street, Liverpool, New South Wales 2170, Australia.

^8^ St Vincent’s Hospital, 390 Victoria Street, Darlinghurst, New South Wales, 2010 Australia.

^9^Westmead Hospital, Hawkesbury and Darcy Roads, Westmead, New South Wales 2145, Australia.

^10^Royal Prince Alfred Hospital, Missenden Road, Camperdown, New South Wales 2050, Australia.

^11^Prince of Wales Hospital, Barker Street, Randwick, New South Wales 2031, Australia.

^12^Fremantle Hospital, Alma Street, Fremantle, Western Australia 6959, Australia.

^13^Sir Charles Gairdner Hospital, Hospital Avenue, Nedlands, Western Australia 6009, Australia.

^14^St John of God Healthcare, 12 Salvado Road, Subiaco, Western Australia 6008, Australia.

^15^Royal Adelaide Hospital, North Terrace, Adelaide, South Australia 5000, Australia.

^16^Flinders Medical Centre, Flinders Drive, Bedford Park, South Australia 5042, Australia.

^17^Envoi Pathology, 1/49 Butterfield Street, Herston, Queensland 4006, Australia.

^18^Princess Alexandria Hospital, Cornwall Street & Ipswich Road, Woolloongabba, Queensland 4102, Australia.

^19^Austin Hospital, 145 Studley Road, Heidelberg, Victoria 3084, Australia.

^20^Johns Hopkins Medical Institute, 600 North Wolfe Street, Baltimore, Maryland 21287, USA.

^21^ARC-NET Center for Applied Research on Cancer, University of Verona, Via dell’Artigliere, 19 37129 Verona, Province of Verona, Italy.

^22^Wolfson Wohl Cancer Research Centre, Institute of Cancer Sciences, University of Glasgow, Garscube Estate, Switchback Road, Bearsden, Glasgow, Scotland G61 1BD, United Kingdom.

^23^Wollongong Hospital, Illawarra and Shoalhaven Local Health District, Loftus Street, Wollongong NSW 2500.
